# Protocol for constructing glycan biosynthetic networks using glycowork

**DOI:** 10.1016/j.xpro.2024.102937

**Published:** 2024-04-16

**Authors:** Jon Lundstrøm, Luc Thomès, Daniel Bojar

**Affiliations:** 1 Department of Chemistry and Molecular Biology, University of Gothenburg, 41390 Gothenburg, Sweden; 2 Wallenberg Centre for Molecular and Translational Medicine, University of Gothenburg, 41390 Gothenburg, Sweden; 3 University Lille, CHU Lille, ULR 7364 - RADEME - Maladies RAres du DÉveloppement embryonnaire et du Métabolisme, 59000 Lille, France

**Keywords:** Bioinformatics, Sequence analysis, Evolutionary biology

## Abstract

Glycans, present across all domains of life, comprise a wide range of monosaccharides assembled into complex, branching structures. Here, we present an *in silico* protocol to construct biosynthetic networks from a list of observed glycans using the Python package glycowork. We describe steps for data preparation, network construction, feature analysis, and data export. This protocol is implemented in Python using example data and can be adapted for use with customized datasets.

For complete details on the use and execution of this protocol, please refer to Thomès et al.^1^

## Highlights

• Steps for environment setup and data preparation
• Instructions for building and plotting biosynthetic networks
• Steps for pruning networks using evolutionary information
• Steps for analysis of network statistics and feature highlighting

## Abstract (editor highlights)

Publisher’s note: Undertaking any experimental protocol requires adherence to local institutional guidelines for laboratory safety and ethics.

## Graphical abstract

**Figure:**
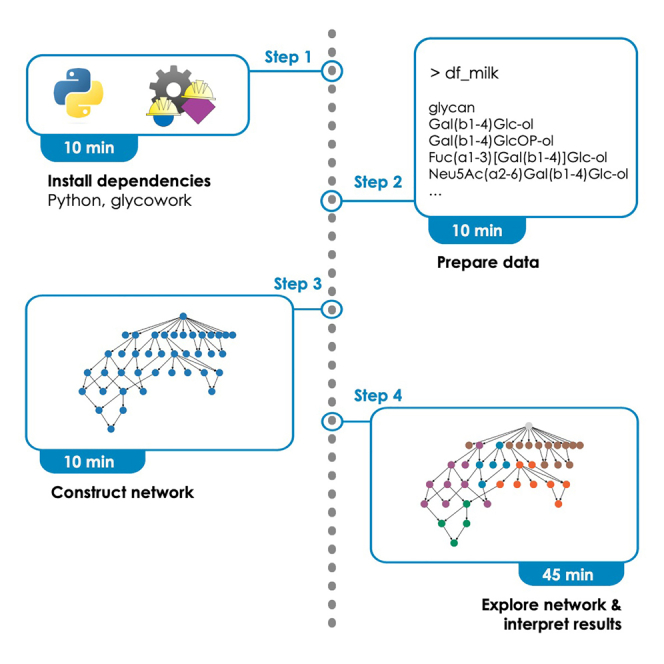

## Before you begin

This protocol provides a detailed walkthrough of how to construct biosynthetic networks based on a known list of related glycan structures. All code necessary to replicate the method presented here has been implemented in a Jupyter notebook format and is available as File S1: **protocol_notebook.ipynb**. The notebook file can be uploaded to a personal Google Drive folder and launched with Google Colaboratory. Alternatively, the notebook can be executed locally using Jupyter Notebook (https://jupyter.org/install). An installation of Python version 3.8 or above (https://www.python.org/downloads/) is necessary for glycowork and thus for running the protocol. We demonstrate the protocol using the milk glycan dataset described in Thomès et al. (2023),^1^ but the protocol can be adapted to any other dataset of interest, including other glycan classes. Importantly, the set of glycans used for network generation should only contain glycans of the same class (e.g., *N*-, *O*-, lipid-linked, or free glycans).

### System requirements

This protocol can be performed using any modern personal computer, as it does not require heavy computational power to run (see limitations for exceptions.) Online computing services, such as Google Colaboratory, are viable alternatives to local processing, and are recommended by the authors due to the ease of setting up the environment and the reproducibility of the notebook format. A Python installation is required for running the code described in this protocol. Necessary packages and publicly available datasets are listed in the key resources table. If desired, exported networks can be further analyzed and edited using specialized software such as Cytoscape or Gephi.

### Installation of glycowork and setup of environment

**Timing: 10 min.**

**CRITICAL:** All code examples in this protocol are intended to be run in a notebook environment such as Google Colaboratory or Jupyter Notebook. If running the code in a regular Python console instead, lines prefaced with a ‘!’ operator need to instead be run in a command line environment (only relevant for the installation of the necessary dependencies).

***Optional:*** To run the notebook code using Google Colaboratory, skip this optional section and continue at Step 1. Alternatively, perform the following steps to install glycowork into a virtual environment in order to run the notebook locally. (i) Download and install Miniconda from https://docs.conda.io/projects/miniconda/en/latest/ according to your computer operating system. (ii) Using the command-line interface of your operating system, run the code below to install glycowork and dependencies into a virtual environment and launch the notebook locally. The files **protocol_notebook.ipynb** and **environment.yml** are available as Files S1 and S2, respectively. (iii) Before running the notebook, comment out the code in Step 1 (as glycowork has already been installed) and modify the code in Step 4 to define an appropriate local file path.

> conda env create -n glycowork -f environment.yml # install virtual environment

> conda activate glycowork # activate virtual environment

> jupyter notebook # launch Jupyter notebook

***Note:*** If running the code outside of a notebook environment, generated figures will not be displayed in-line.

1. Install glycowork. (Troubleshooting 1).

> !pip install glycowork==1.0.1 # delete '!' operator if running in Python console

***Optional:*** If using the built-in module for drawing glycan structures using GlycoDraw,^2^ the glycowork install needs to be performed as below.

***Note:*** If not working in a Google Colaboratory environment, see the GlycoDraw section (https://bojarlab.github.io/glycowork/examples.html#glycodraw-code-snippets) of the glycowork documentation for installation instructions.

> !pip install glycowork[draw]==1.0.1 # delete '!' operator if running in Python console

2. Import all necessary helper functions from glycowork.

> import glycowork

> from glycowork.glycan_data.loader import df_glycan, build_custom_df, unwrap

> from glycowork.motif.processing import get_lib

> from glycowork.motif.graph import generate_graph_features

> from glycowork.network.biosynthesis import construct_network, plot_network, export_network, highlight_network, trace_diamonds, find_diamonds, network_alignment, infer_virtual_nodes, infer_network, retrieve_inferred_nodes, prune_network, evoprune_network

> from glycowork.network.evolution import jaccard, get_communities, calculate_distance_matrix

3. Import additional dependencies.

> import matplotlib

> import matplotlib.pyplot as plt

> import matplotlib.patheffects as path_effects

> #comment out below line if running in Python console

> %matplotlib inline

> from scipy.stats import ttest_ind

> import seaborn as sns

> import pandas as pd

> import numpy as np

> import networkx as nx

> import pickle

***Note:*** These dependencies are bundled with glycowork or come pre-installed when working in a Google Colaboratory environment. When working in a local Python console, install (e.g., *pip install ...*) the necessary packages prior to importing.

4. Set the working file path, assuming the presence of a folder named “glycan_networks” in Google Drive.

> fp = 'drive/My Drive/glycan_networks/'

5. Download the milk glycan data from Thomès et al. (2023)^1^ and place in the “glycan_networks” folder. All data is available as supplementary data or through Zenodo (https://zenodo.org/records/8016366). If downloading from Zenodo, unzip “MilkGlycans-archived.zip” and locate following files in BojarLab-MilkGlycans-17c6650/data.
  a. “TableS2.csv” contains milk glycan sequences in IUPAC-condensed format and associated species annotations.
  b. “milk_networks_exhaustive.pkl” contains pre-calculated milk glycan biosynthetic networks.

## Key resources table

**Table:** 

REAGENT or RESOURCE	SOURCE	IDENTIFIER
**Deposited data**		

Milk oligosaccharide dataset	Thomès et al.^1^	https://zenodo.org/record/8016366 ‘TableS2.csv’
Precomputed milk oligosaccharide networks	Thomès et al.^1^	https://zenodo.org/record/8016366 ‘milk_networks_exhaustive.pkl’
SugarBase	Thomès et al.^3^	https://zenodo.org/records/10259832 ‘BojarLab-glycowork-40b04d7/glycowork/glycan_data/v9_sugarbase.pkl’

**Software and algorithms**		

Python 3.10.12	https://www.python.org/	https://www.python.org/
glycowork 1.0.1	Thomès et al.^3^	https://zenodo.org/records/10259832 https://github.com/BojarLab/glycowork
Matplotlib 3.7.1	Hunter et al.^4^	https://matplotlib.org/
SciPy 1.11.4	Virtanen et al.^5^	https://scipy.org/
Seaborn 0.12.2	Waskom^6^	https://seaborn.pydata.org/
pandas 1.5.3	McKinney^7^	https://pandas.pydata.org/
NumPy 1.23.5	Harris et al.^8^	https://numpy.org/
NetworkX 3.2.1	Hagberg et al.^9^	https://networkx.org/
pickle 4.0	https://www.python.org/	https://docs.python.org/3/library/pickle.html
Google Colaboratory	https://research.google/	https://research.google.com/colaboratory/

## Step-by-step method details

### Preparation of data

**Timing: 10 min**

This step shows how to prepare the example data for biosynthetic network generation.

1. Load the table containing milk glycan structures and associated species information. (Troubleshooting 2).

> df_milk = pd.read_csv(fp + 'TableS2.csv')

2. Prepare library of glycoletters and list of species from the dataset.

> lib = get_lib(df_milk.target.values.tolist())

> species_list = list(sorted(list(set(df_milk.Species.values.tolist()))))

3. The final section of the protocol, *Building networks with other glycan types*, requires a collection of related glycans to be provided as input for the network generation. Here, glycans are prepared by loading and filtering SugarBase.
  ***Note:*** The SugarBase database was introduced with the release of glycowork^3^ and remains continuously maintained and updated. While the current protocol demonstrates the construction of biosynthetic networks using glycans extracted from SugarBase, data from in-house experiments or from other databases such as GlyGen^10^ or GlyTouCan^11^ can be utilized as well. Important considerations when applying the protocol to a new dataset are outlined in the Limitations section.
  a. Build the SugarBase glycan table containing sequence information with associated metadata.
    > df_species = build_custom_df(df_glycan)
  b. Save a copy of the table, filtered to contain only glycolipids.
    > df_glycolipid = build_custom_df(df_glycan[df_glycan['glycan_type']=='lipid'])
  c. Save a copy of the table, filtered to contain only *O*-glycans.
    > df_oglycans = build_custom_df(df_glycan[df_glycan['glycan_type']=='O'])

### Build network with inferred intermediate structures

**Timing: 10 min**

This step describes how to select a subset of milk glycans from a given species, and how to build and plot the corresponding biosynthetic network.

4. Create a species-specific DataFrame from the full dataset.

***Note:*** Here, the species *Lama pacos* is used as an example. In the code, *'Lama_pacos'* can be replaced by another species of interest. Available species are stored in the *species_list* variable previously defined.

> df_lama = df_milk[df_milk.Species == 'Lama_pacos'].reset_index(drop=True)

5. Build a glycan network. (Troubleshooting 3).

> net_lama = construct_network(df_lama.target.values.tolist(), libr = lib)

6. Plot the computed network. (Troubleshooting 4).

> plot_network(net_lama)

### Prune network

**Timing: 10 min.**

This step allows pruning of a given network by comparing it to other processed networks.

***Note:*** Evolutionary pruning requires multiple networks stored in a dictionary. Here, we use pre-calculated milk networks from Thomès et al. (2023)^1^, obtained in step 5 of the *Before you begin – Installation of glycowork and setup of environment* section.

7. Load the dictionary containing pre-calculated milk networks.

> with open(fp + 'milk_networks_exhaustive.pkl', 'rb') as file:

> net_dic = pickle.load(file)

***Note:*** The *net_dic* dictionary is structured with species as keys and network objects of glycan networks as values. If desired, a similar network dictionary can be prepared by updating an existing dictionary object with the appropriate key-value pairs (i.e., *custom_net_dic[‘key_label’] = network_object*).

8. Prune the network from the previous example.

> net_lama2 = evoprune_network(net_lama, network_dic = net_dic, species_list=species_list, libr = lib, threshold = 0.01)

***Note:*** During pruning, alternative reaction paths are compared across all networks and a path is pruned if a significant difference in path likelihood (default *threshold*, p < 0.01) is observed. For more details, see Thomès et al. (2023).^1^ The *threshold* value can be modified to change the stringency of the statistical comparison, and thus, the network pruning.

9. Plot the pruned network.

> plot_network(net_lama2)

### Analyzing network features

**Timing: 10 min**

This step describes how to compute multiple network statistics and how to compare previously generated or loaded networks.

10. Plot node degrees.
  a. Compute each node’s degree.
    > degree = net_lama2.degree()
    > degree_list = []
    > for (n,d) in degree:
    > degree_list.append(d)
  b. Plot the results in a histogram.
    > plt.hist(degree_list)
    > plt.title('Lama pacos pruned network degree')
    > plt.xlabel('Node degree')
    > plt.ylabel('Occurrence')
11. Collect general network features.

> # For a single network:

> features = generate_graph_features(net_lama2.to_undirected(), glycan_graph = False, libr = lib, label = 'Lama_pacos')

> # For multiple networks extracted from a dictionary:

> all_networks = [net_dic[k].to_undirected() for k in species_list]

> feats = []

> for k in range(len(all_networks)):

> try:

> spec = species_list[k]

> feats.append(generate_graph_features(all_networks[k], glycan_graph = False, libr = lib, label = spec))

> except:

> pass

> df_feat = pd.concat(feats, axis=0)

***Note:*** The *generate_graph_features()* function requires an undirected network graph object (converted with *net_lama2.to_undirected()*) as input. (Troubleshooting 5). An overview of function parameters can be found in the glycowork documentation pages (https://bojarlab.github.io/glycowork/).

***Note:*** See Table S1**: General network features** for a full overview of the resulting table.

12. Compare inferred versus observed node degree across networks.

> collecty = []

> for net in all_networks:

> node_state = list(nx.get_node_attributes(net, 'virtual').values())

> degree_state = list(net.degree())

> degree_state = [k[1] for k in degree_state]

> collecty.append(list(zip(node_state, degree_state)))

> collecty = unwrap(collecty)

> # Removing root and terminal nodes

> collecty = [k for k in collecty if k[1]>1]

> # Plotting

> temp = pd.DataFrame(collecty)

> temp.columns = ['virtual','degree']

> ax = sns.boxplot(x="virtual", y='degree', data=temp)

> plt.title('observed versus virtual node degree')

***Note:*** Inferred nodes are referred to as “virtual” nodes in the code. This attribute is encoded as a binary label: 0 – experimentally observed node, 1 – inferred/virtual node.

***Optional:*** Statistical tests can be performed to compare a given feature between observed and virtual nodes using the SciPy module, such as here with a one-sided Welch’s t-test:

> virtuals = [k[1] for k in collecty if k[0] == 1]

> reals = [k[1] for k in collecty if k[0] == 0]

> ttest_ind(virtuals, reals, alternative = 'greater', equal_var = False)[1]

***Note:*** With a calculated p-value of p < 10^−9^, there is a statistically significant difference between the average node degree of observed and virtual nodes.

13. Extract biosynthetic modules of glycan communities from a single network and highlight in the original network figure.

> # get communities

> lama_comms = get_communities([net_lama2.to_undirected()])

> # build networks from

> all_comm_networks = []

> for k in lama_comms.keys():

> comm_network = construct_network(lama_comms[k], libr = lib)

> all_comm_networks.append(comm_network)

> merged_network = nx.compose_all(all_comm_networks)

# get color map

col = ['#058F60', '#0385AE', '#A15989', '#9F6D55', '#EF6130', '#C23537']

color_dic = {}

for k in range(len(lama_comms.keys())):

for j in lama_comms[list(lama_comms.keys())[k]]:

color_dic[j] = col[k]

# set gray color for nodes occurring in multiple communities

for k in set([k for k in unwrap(list(lama_comms.values())) if unwrap(list(lama_comms.values())).count(k) > 1]):

color_dic[k] = '#D3D3D3'

> # draw network

> nx.draw(net_lama2, pos = nx.nx_pydot.pydot_layout(net_lama2, prog = "dot"), node_color = [color_dic[k] for k in list(net_lama2.nodes())])

***Note:*** Like *generate_graph_features()*, the *get_communities()* function requires an undirected networkx graph as input.

***Note:*** The *get_communities()* function returns a merged dictionary of community : glycans in that community.

14. Calculate communities across all milk networks and calculate pairwise Jaccard distances for clustering.

> all_networks = [net_dic[k].to_undirected() for k in species_list]

> comms = get_communities(all_networks, label_list = species_list)

> comms = {k:v for k,v in comms.items() if v != ['Gal(b1-4)Glc-ol']}

> dm = calculate_distance_matrix(comms, jaccard)

***Note:*** Communities only consisting of lactose are removed as these originate from understudied species where only lactose has been observed.

***Note:*** Runtime scales with network complexity.

15. Compare extracted communities by clustering.

> idx = [k for k in range(len(dm)) if np.mean(dm.iloc[:,k].values.tolist())<0.96]

> dm2 = dm.iloc[idx, idx].reset_index(drop=True)

> sns.clustermap(dm2)

### Editing and exporting networks in glycowork

**Timing: 10 min**

This step describes how to edit networks to highlight select features using built-in glycowork features.

16. Highlighting network conservation by scaling of node size.
  a. Construct and prune biosynthetic network using milk glycans from the Diprotodontia order.
    > df_diproto = df_milk[df_milk.Order == "Diprotodontia"].reset_index(drop=True)
    > net_diproto = construct_network(list(set(df_diproto.target.values.tolist())), libr = lib)
    > net_diproto2 = evoprune_network(net_diproto, net_dic, species_list = species_list, libr = lib, threshold = 0.01)
    ***Note:*** The *df_milk* table contains glycan information from 172 unique mammalian species. While we demonstrate highlighting of network features using glycans from the Diprotodontia order, we encourage users to explore the dataset and modify the code to build and annotate different biosynthetic networks.
  b. Highlight network conservation across species of the Diprotodontia order.

> net_diproto2 = highlight_network(net_diproto2, highlight = 'conservation', conservation_df = df_milk[df_milk.Order == 'Diprotodontia'])

> plot_network(net_diproto2)

***Note:*** The *highlight_network()* function allows for highlighting of different attributes, including glycan motifs, glycan abundance, glycan conservation (as shown in the above example), and species (for highlighting one species in a multi-species network). See the documentation (https://bojarlab.github.io/glycowork/network.html#highlight_network) for further details.

17. Here, files including information on connectivity and annotation are exported for further modeling and visualization using external software.

> export_network(net_lama2, filepath = fp + 'lama_export')

***Note:*** The *filepath* argument should describe a valid path and file name prefix which will be appended with file description and type upon saving.

***Note:*** The *export_network()* function generates two files: “prefix_node_labels.csv” containing observed/virtual node label information and “prefix_edge_list.csv” containing connectivity information. The files generated from this function call are included in Table S2: Exported network files.

***Note:*** The exported files can be imported into external graph software, such as Gephi or Cytoscape, for further annotation.

### Building networks with other glycan types

**Timing: 10 min**

This step describes how other glycan types than milk glycans can be used to generate networks, including glycolipids and *O*-glycans.

18. Create network from zebrafish glycolipids.

> glycolipid_zebrafish = df_glycolipid['glycan'][df_glycolipid['Species'].str.contains('Danio_rerio')==True].to_list()

> lib = get_lib(glycolipid_zebrafish)

> net_zebrafish = construct_network(glycolipid_zebrafish, libr = lib)

> plot_network(net_zebrafish)

19. Create network from chicken *O*-glycans.

> oglycan_chicken = df_oglycans['glycan'][df_oglycans['Species'].str.contains('Gallus_gallus')==True].to_list()

> lib = get_lib(oglycan_chicken)

> net_chicken = construct_network(oglycan_chicken, libr = lib)

> plot_network(net_chicken)

## Expected outcomes

**Figure 1:**
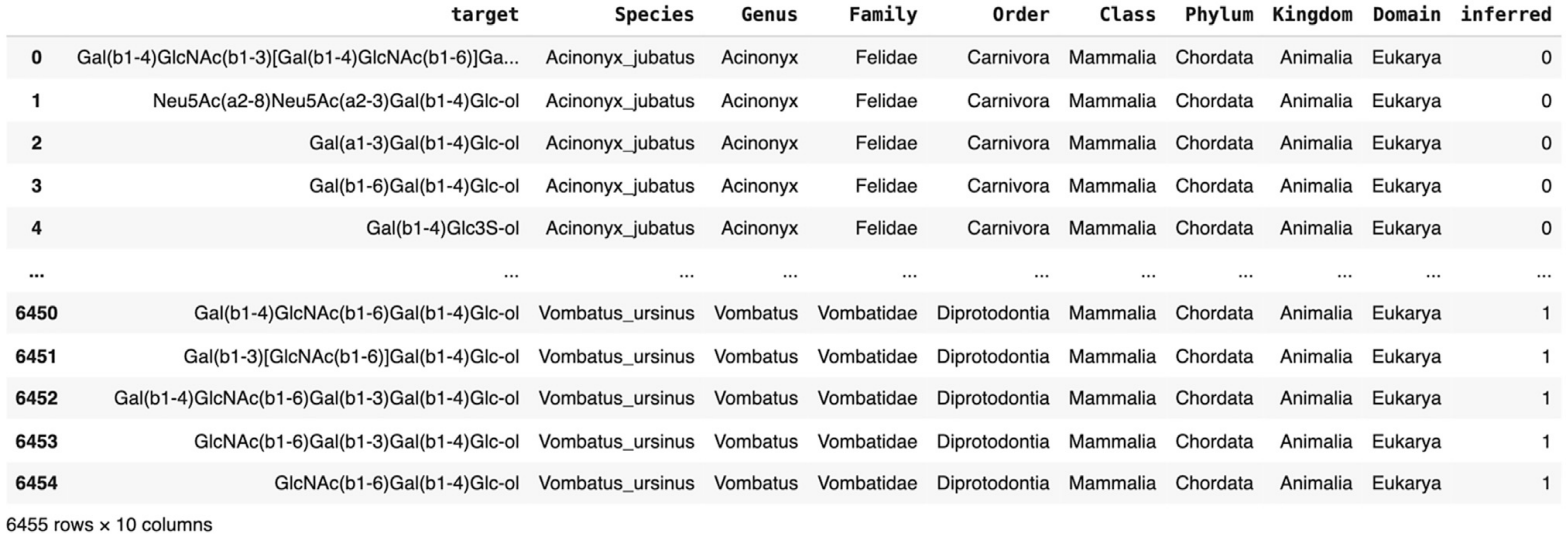
Screenshot of TableS2.csv (df_milk) This table contains milk glycan structures and associated species information.

**Figure 2:**
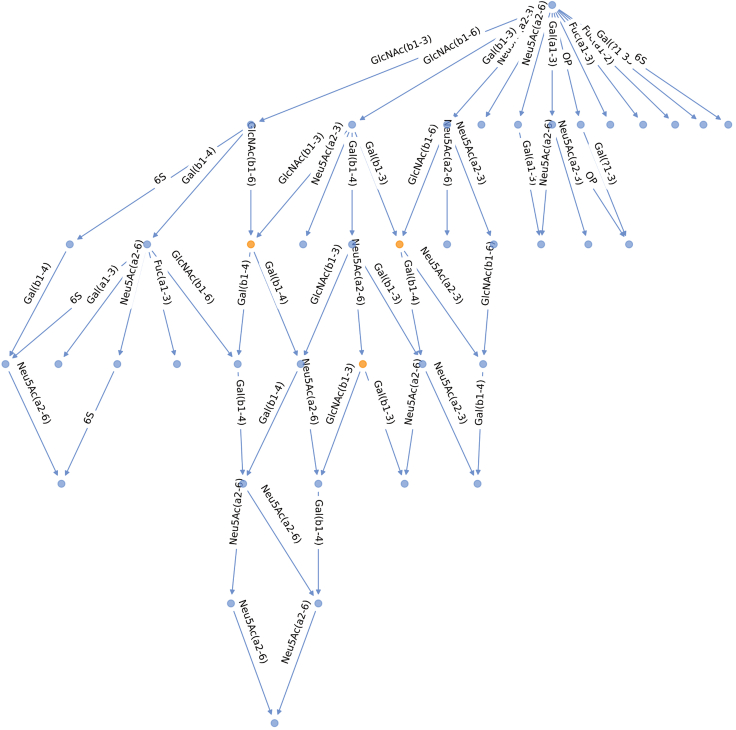
Biosynthetic network of *Lama pacos* milk glycans Nodes represent glycan structures (blue – observed, orange – inferred). The milk glycan lactose core (Galβ1-4Glc) is located at the top of the network. Edges indicate the enzymatic reaction, i.e., the addition of a monosaccharide or chemical modification, separating two nodes. Viewing of the generated network in a notebook format allows for mouse-over of nodes to get the identity of the structure.

**Figure 3:**
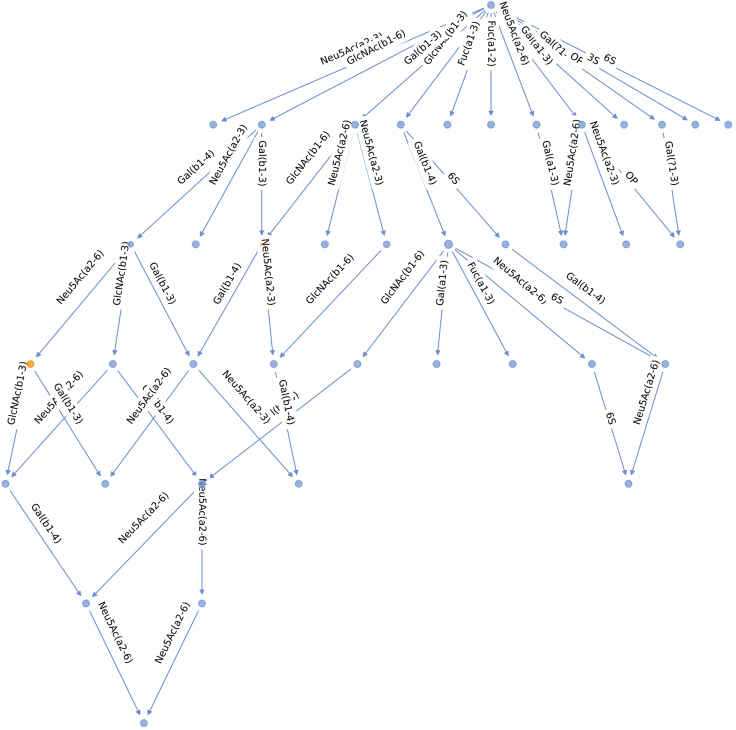
Pruned biosynthetic network of *Lama pacos* milk glycans Glycan structures and enzymatic reaction steps are represented as described in Figure 2.

**Figure 4:**
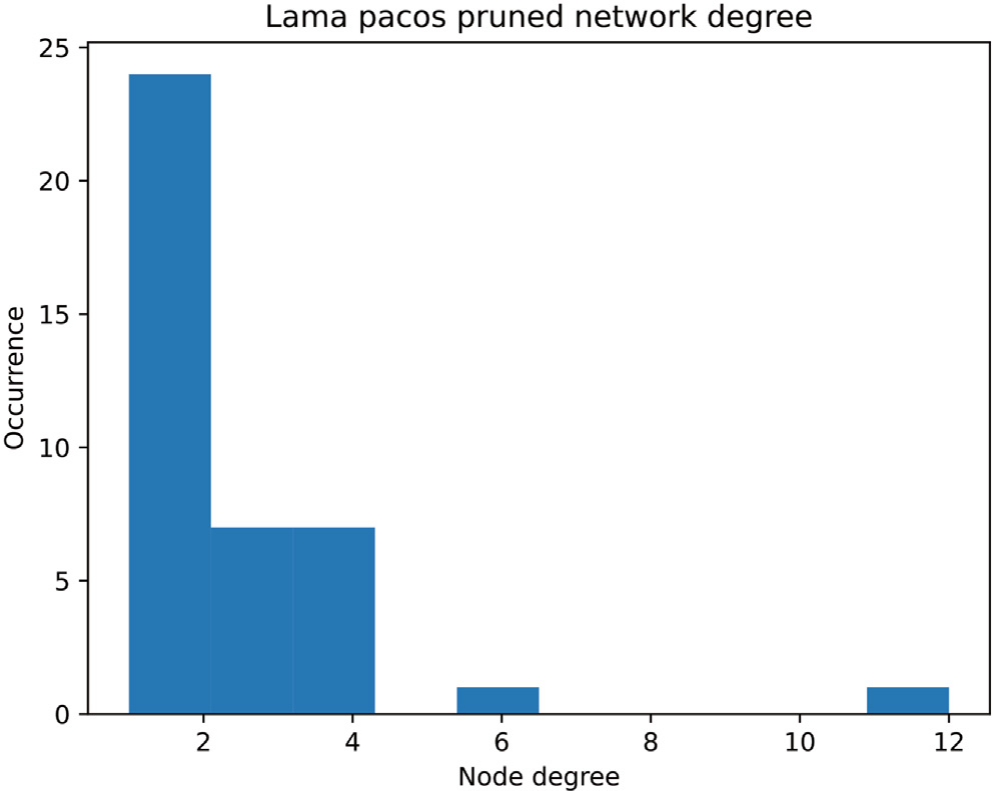
Pruned *Lama pacos* network statistics Degree indicates the number of total connections a node exhibits. Node degree analysis yields insights into overall network connectivity and can provide hypotheses for biosynthetic flux of intermediate structures.

**Figure 5:**
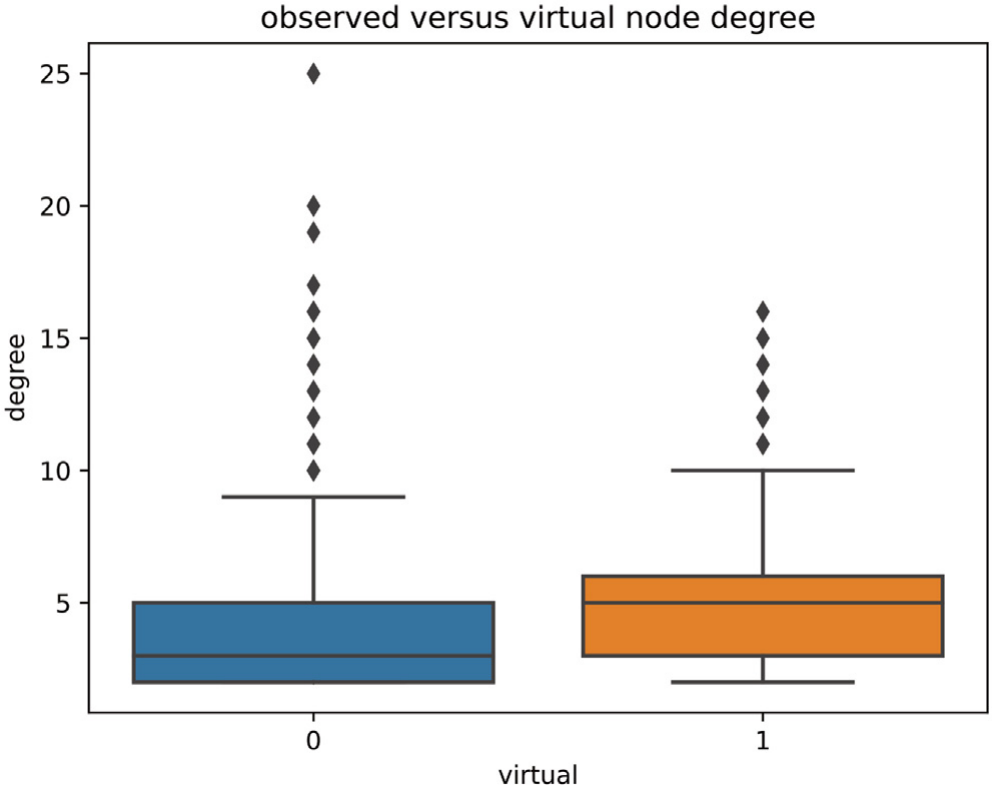
Comparison of node degree statistics between observed and virtual nodes across the full dataset Data are depicted as mean values, with box edges showing quartiles and whiskers representing the remaining data distribution. Data points outside the whiskers are outliers and marked with dots.

**Figure 6:**
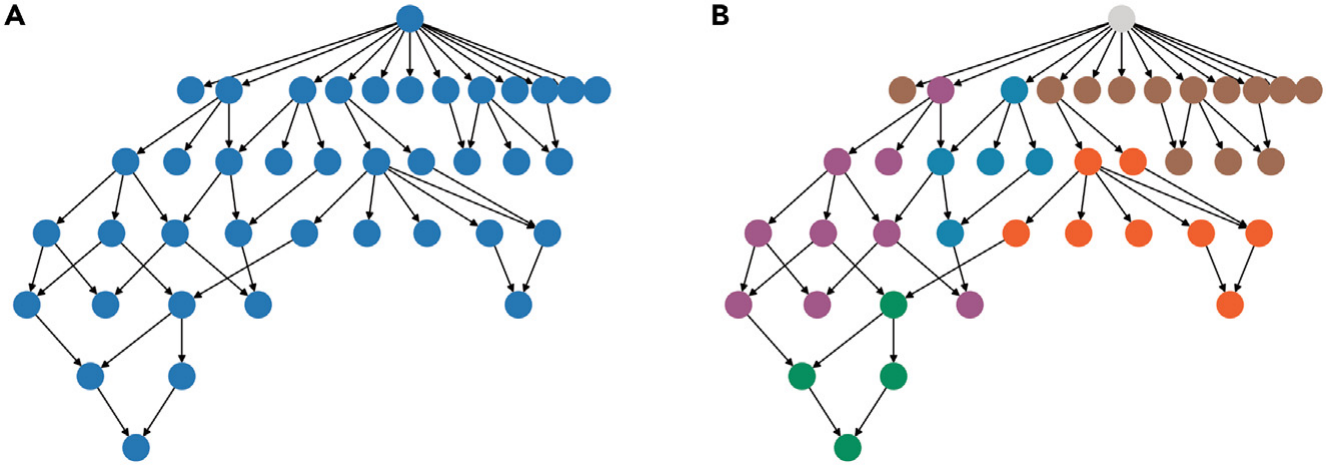
*Lama pacos* network communities (A) Pruned *Lama pacos* network. (B) Pruned *Lama pacos* network with node colors indicating community identity. Nodes overlapping multiple communities are colored in light gray.

**Figure 7:**
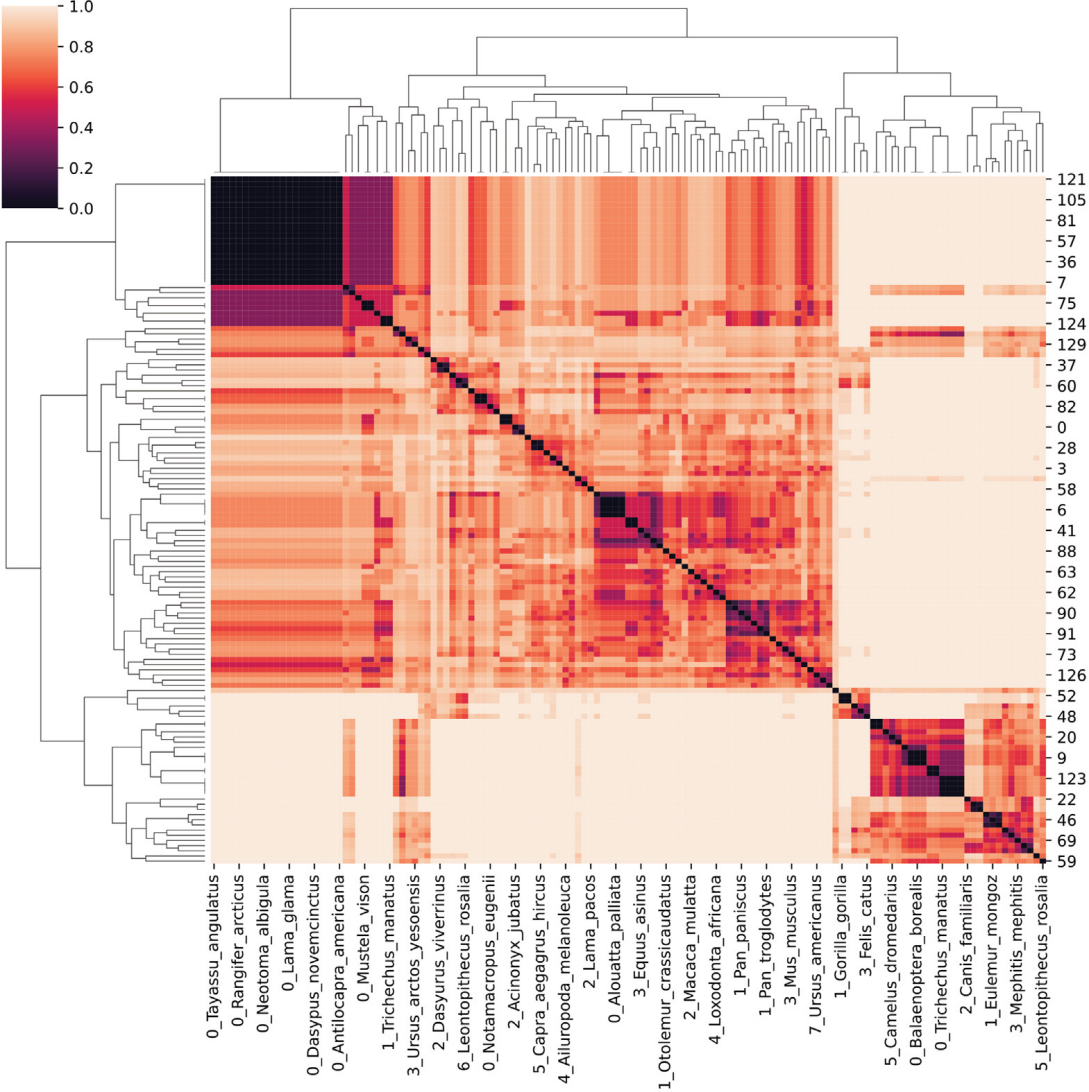
Hierarchical clustering of pairwise Jaccard distances between communities across the full dataset The clustering reveals three highly conserved biosynthetic modules containing (i) *N*-acetyllactosamine-based MOs, (ii) MOs derived from the progenitor GlcNAcβ1-3Galβ1-4Glc, and (iii) MOs starting from the progenitor GlcNAcβ1-6Galβ1-4Glc. Further insights into these three modules are discussed in Thomès et al. (2023).^1^

**Figure 8:**
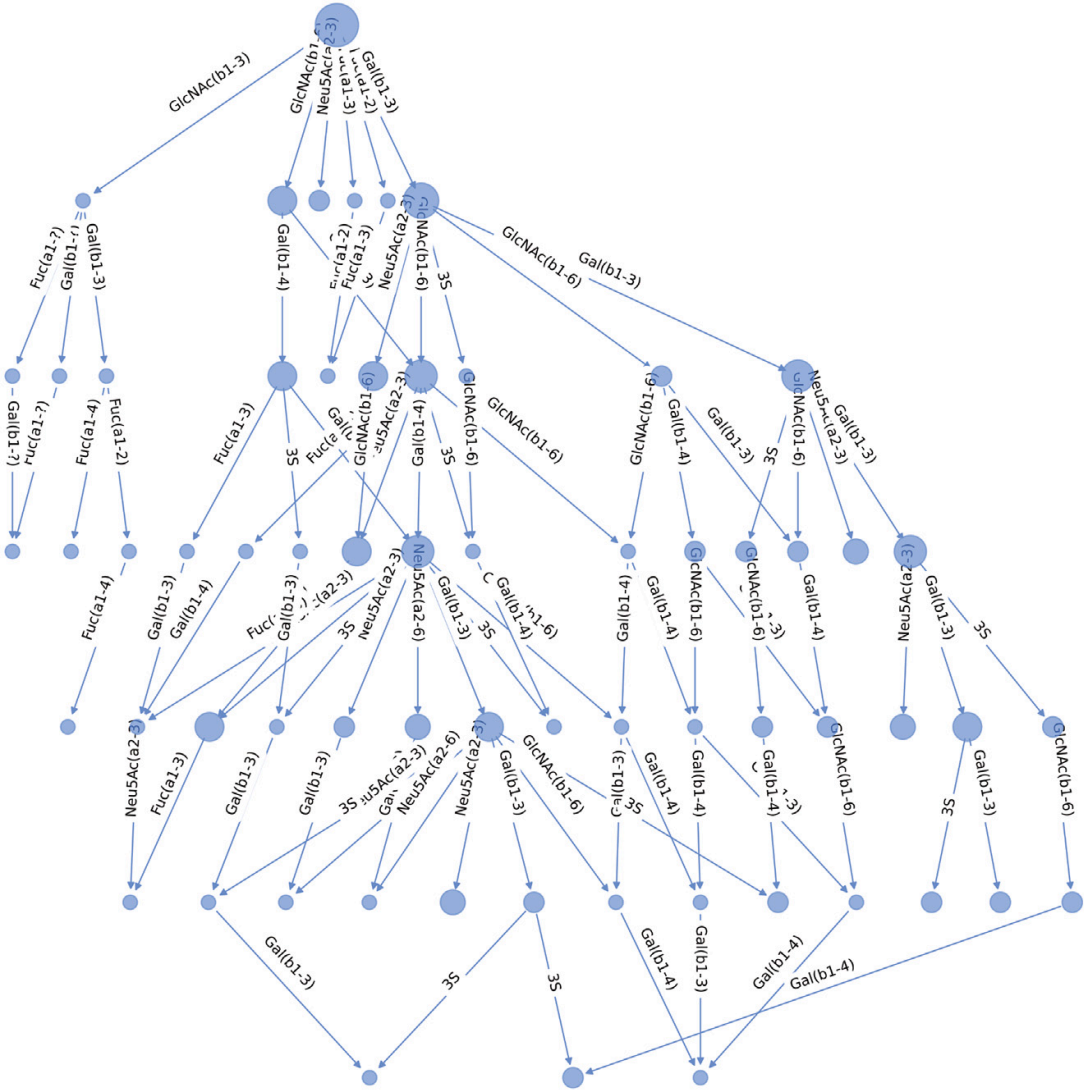
Biosynthetic network of milk oligosaccharides from the Diprotodontia order Glycan structures and enzymatic reaction steps are represented as described in Figure 2. Node size is scaled by degree of conservation across the order of Diprotodontia.

**Figure 9:**
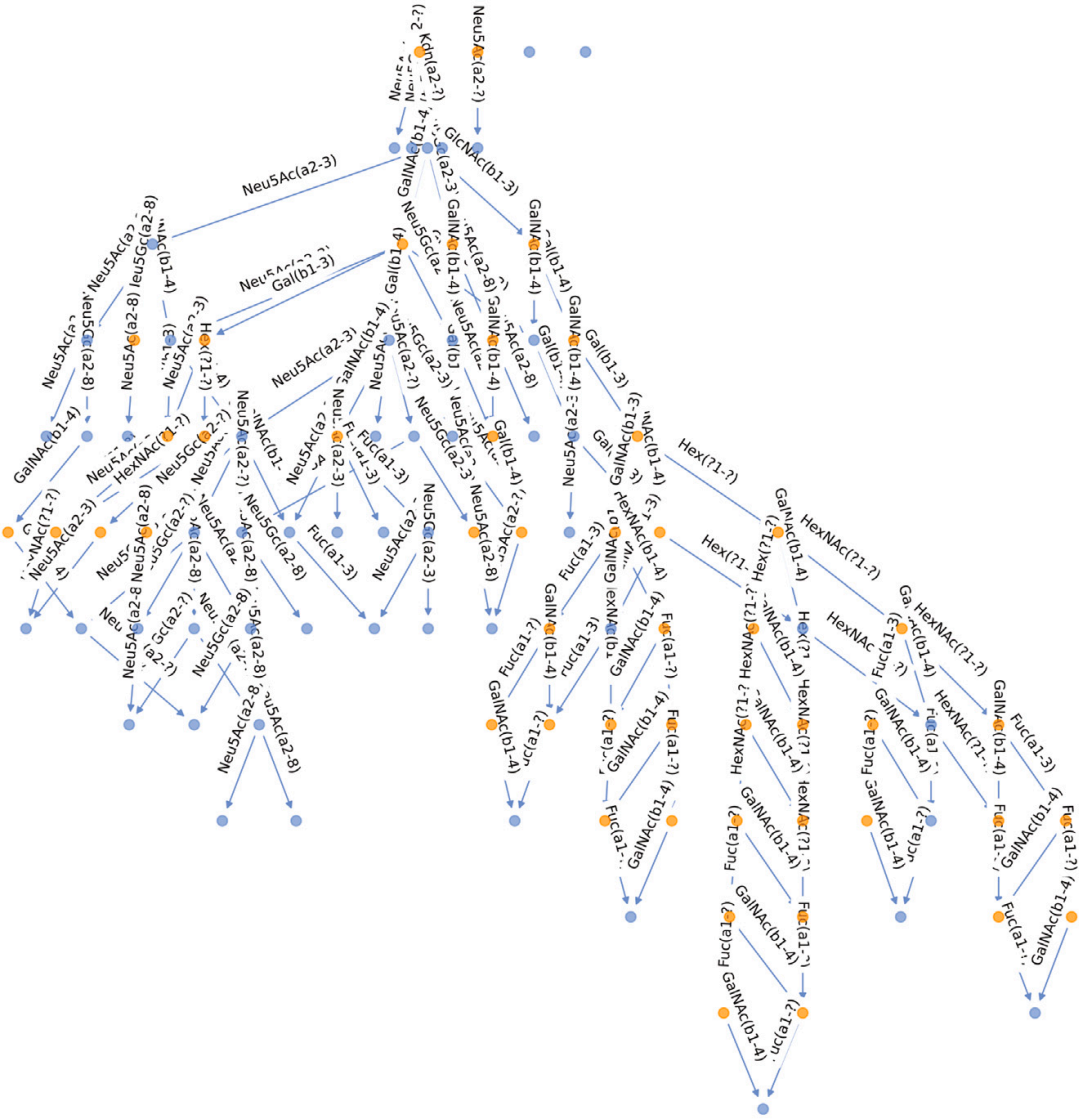
Biosynthetic network of zebrafish glycolipids Glycan structures and enzymatic reaction steps are represented as described in Figure 2. Multiple network roots represent the Glcβ1Cer glycolipid core (top left) and structures that cannot be connected to the rest of the network in five or fewer inferred intermediate steps.

**Figure 10:**
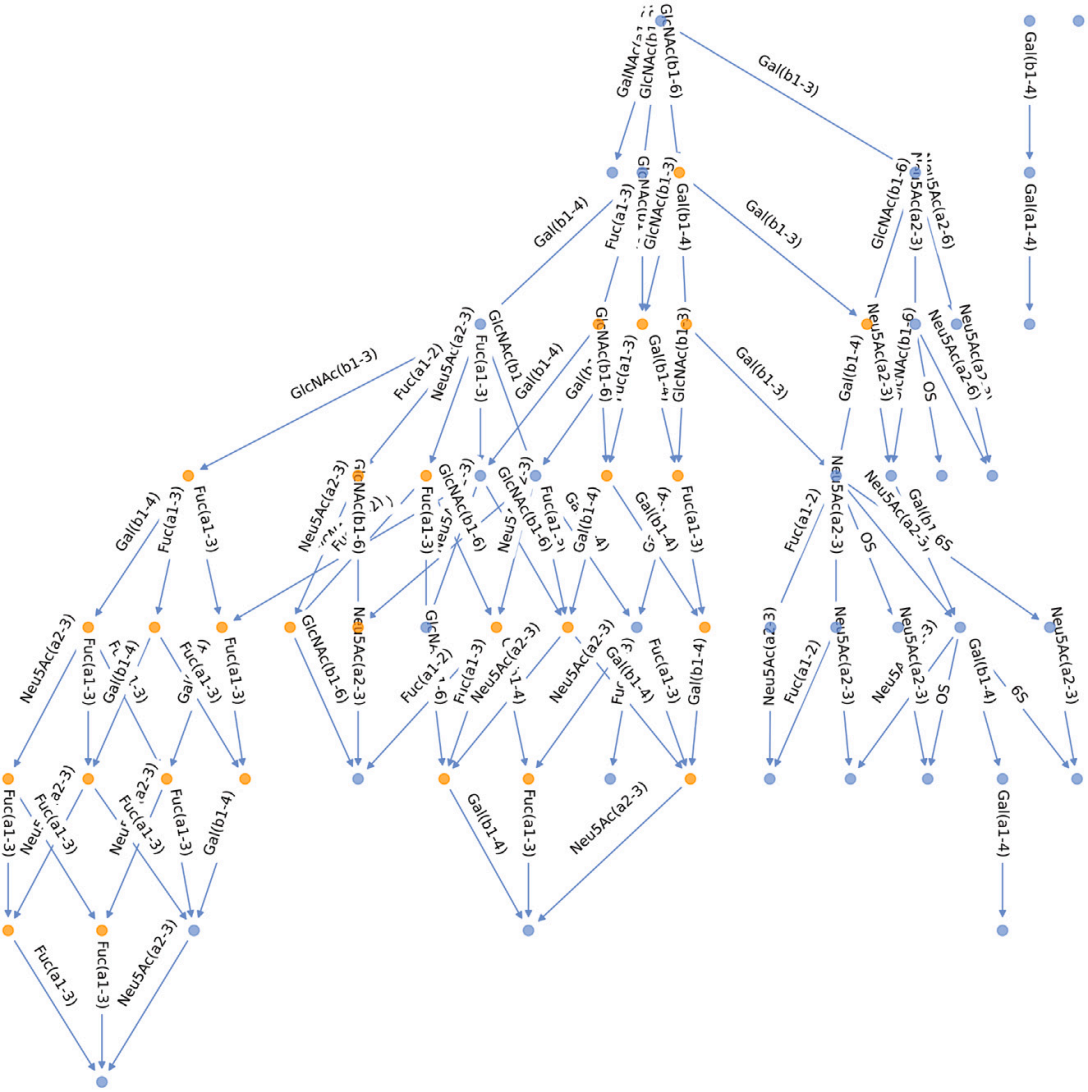
Biosynthetic network of chicken *O*-glycans Glycan structures and enzymatic reaction steps are represented as described in Figure 2. Multiple network roots represent GalNAc (top left) and structures that cannot be connected to the rest of the network in five or fewer inferred intermediate steps. Diverging branches of the main network represent extension of distinct *O*-glycan core structures.

Successfully running the protocol will result in the generation of a list of files and figures. In steps 1–6, an initial biosynthetic network for milk glycans from *Lama pacos* is generated (Figures 1 and 2). In steps 7–10, this network is further pruned using evolutionary information obtained from the full dataset (Figure 3). Steps 10–15 describe the analysis of network features, including node degree (Figures 4 and 5), general network statistics (Table S1), and community detection (Figures 6 and 7). Step 16 showcases how to highlight network features within glycowork (Figure 8), while step 17 allows export of network files for further analysis and annotation using external software (Table S2). Finally, steps 18–19 give examples of network generation using alternative glycan classes (Figures 9 and 10).

## Limitations

As different glycan types are synthesized through different pathways, a network can only connect glycans from the same type. Therefore, any attempt to construct a network from a heterogeneous list of glycan types will result in a failure of this protocol.

In addition to the available computational power, two factors are strongly influencing the run time when constructing a network: the number of glycans in the initial list, and the maximum metabolic distance between these oligosaccharides. While the first reason may sound obvious, as more glycans will require more calculations and constitute additional entities to handle, the second reason is less straightforward. If few glycans are observed and if they are separated by many biosynthetic steps, meaning that they are of very heterogeneous length and/or composition, computing all possible intermediates to generate a single network will be a more fastidious task, ultimately resulting in a longer run time. To counteract that, glycowork will not try to connect parts of the network that have more than five missing intermediates in a row (i.e., it would take more than five reactions to connect a large observed structure to any smaller observed structure). This may sometimes result in incompletely connected networks.

## Troubleshooting

### Problem 1

Failure to install glycowork (Before you begin – Installation of glycowork and setup of environment).

### Potential solution

Check the version of Python installed.

In notebook:

> !python --version

In console.

> python --version

glycowork requires Python >= 3.8. If the installed version of Python does not meet this requirement, please refer to https://www.python.org/downloads/for download and installation of the latest version.

### Problem 2

Failure to load file (step-by-step method details – Preparation of data).

### Potential solution

It is necessary to mount Google Drive in order to access files through Google Colaboratory.

> from google.colab import drive

> drive.mount('/content/drive')

Make sure that the *fp* variable is correctly defined (default: *fp = 'drive/My Drive/glycan_networks/'*) and that all necessary files are placed in a folder named “glycan_networks” in Google Drive. If running the notebook locally, the *fp* variable must be edited to point to a local folder containing the necessary files.

### Problem 3

Failure to generate network (step-by-step method details – Build network with inferred intermediate structures).

### Potential solution

Check the list of glycans used for network generation and filter if necessary. The *construct_network()* function expects a set of related glycan structures, all belonging to the same class. Further, a very large set of glycans and/or a set of several glycans separated by many biosynthetic steps may result in prohibitively long run times depending on the available processing power.

### Problem 4

Failure to plot network (step-by-step method details – Build network with inferred intermediate structures).

### Potential solution

The *plot_network()* function requires the optional networkx dependencies *pydot* and *graphviz* to be installed.

> conda install graphviz

> pip install pydot

### Problem 5

Related to Step 11 in step-by-step method details – Analyzing network features. The *generate_graph_features()* function will return the error *NetworkXNotImplemented: not implemented for directed type* if an unmodified network graph object from construct_network() is used as input.

### Potential solution

The *generate_graph_features()* function requires an undirected networkx graph object as input. Convert the network object with *network_object.to_undirected().*

## Resource availability

### Lead contact

Further information and requests for resources and reagents should be directed to and will be fulfilled by the lead contact, Daniel Bojar (daniel.bojar@gu.se).

### Technical contact

Technical questions on executing this protocol should be directed to and will be answered by the technical contact, Jon Lundstrøm (jon.lundstrom@gu.se).

### Materials availability

This study did not generate new unique reagents.

### Data and code availability

• Data curated or generated here can be found in the supplementary tables as well as stored as internal datasets within glycowork. Publicly available datasets used are listed in the key resources table.
• All relevant code to run the protocol described here and generate included figures can be found implemented in a notebook format as supplementary data.
• Any additional information required to reanalyze the data reported in this paper is available from the lead contact upon request.

## Supplemental information

File S1. protocol_notebook.ipynb (related to Before you begin & Step-by-step method details)

File S2. environment.yml (related to Before you begin - Installation of glycowork and setup of environment)

Table S1. General network features (related to Step-by-step method details – Analyzing network features)

Table S2. Exported network files (related to Step-by-step method details – Editing and exporting networks in glycowork)
